# An mHealth App for Users with Dexterity Impairments: Accessibility Study

**DOI:** 10.2196/mhealth.9931

**Published:** 2019-01-08

**Authors:** Daihua Yu, Bambang Parmanto, Brad Dicianno

**Affiliations:** 1 Health & Rehab Informatics Department of Health Information Management University of Pittsburgh Pittsburgh, PA United States; 2 Department of Physical Medicine and Rehabilitation University of Pittsburgh Pittsburgh, PA United States

**Keywords:** accessibility, dexterity impairments, disability, mHealth, self-management, smartphone apps, spina bifida, spinal cord injury, wellness, mobile phone

## Abstract

**Background:**

A mobile health (mHealth) system called iMHere (interactive mobile health and rehabilitation) was developed to support individuals with chronic conditions and disability in their self-management regimens. The initial design of iMHere, however, lacked sufficient accessibility for users with a myriad of dexterity impairments. The accessibility of self-management apps is essential in ensuring usability.

**Objective:**

This study aims to increase the usability of the iMHere system for users with dexterity impairments by increasing the app’s accessibility.

**Methods:**

We targeted the accessibility redesign by focusing on the physical presentation and the navigability of the iMHere apps. Six participants presenting with dexterity impairments were included in the usability study of the original and redesigned apps.

**Results:**

We observed a lower number of touches needed to complete tasks (*P*=.09) and time to complete individual tasks (*P*=.06) with the redesigned app than with the original app; a significantly lower time for users to complete all tasks (*P*=.006); and a significantly lower error rate (*P*=.01) with the redesigned app than with the original app. In fact, no errors occurred with use of the redesigned app. Participant-reported overall average usability of the redesigned app (*P*=.007) and usability of individual modules (*P*<.001) were significantly higher than that of the original app due mostly to better ease of use and learnability, interface quality, and reliability.

**Conclusions:**

Improved usability was achieved using a redesigned app. This study offers insight into the importance of personalization in enhancing the accessibility and also identifies strategies for improving usability in app development.

## Introduction

Mobile health (mHealth) technologies, an emergent form of treatment support, offer a variety of health services and information through mobile devices such as phones and tablets [1,2]. Using mobile devices to wirelessly link remote and highly mobile populations, mHealth links users directly with health care providers and systems. Mobile apps have become a popular mode for delivering reminders to conduct self-management activities, collect data, and provide treatment support [3], all with the goal of encouraging behavioral changes and improving health care delivery [4-6]. Some specific self-management techniques include frequent communication between patients and clinicians, as well as continuous adherence to, and adjustment of, complex treatment regimens [7].

Improving users’ self-management skills is of critical importance for improving health outcomes and fostering independent living in persons with disabilities (PwDs) [8-10]. This is especially true for individuals with conditions such as spina bifida (SB) and spinal cord injuries (SCI)—a population of 442,000 in the United States—because these individuals are susceptible to secondary complications such as urinary tract infections, constipation, skin breakdown (due to paralysis and loss of sensation), and sepsis [11-13]. These secondary complications are, in part, preventable, but this requires active involvement on the part of patients, caregivers, and clinicians in adherence to self-management regimens. Therefore, developing technologies that promote self-management skills in this population could have a profound impact on health outcomes.

**Figure 1:**
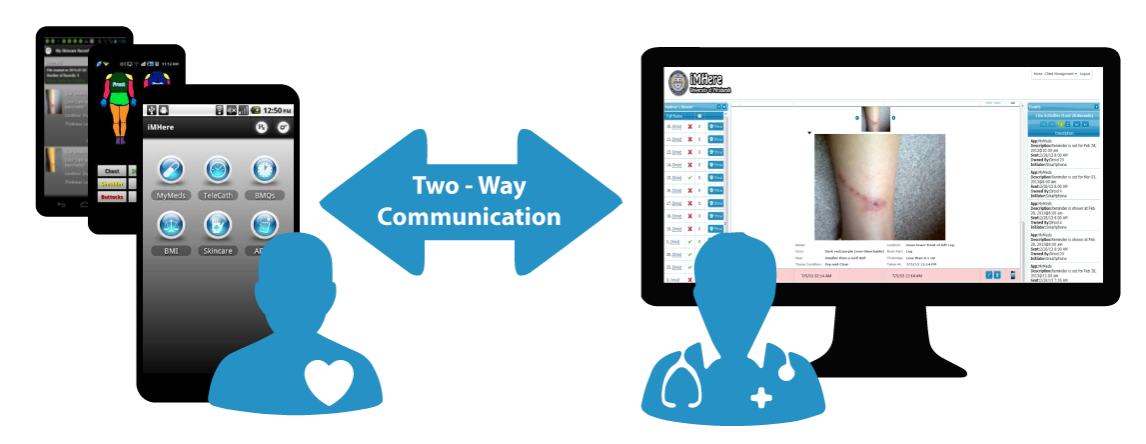
Architecture of the iMHere (interactive mobile health and rehabilitation) system.

Investigators at the University of Pittsburgh have developed a novel mHealth system aimed at empowering persons with chronic conditions like SB and SCI and clinicians to be engaged in improving patient health [14]. This mHealth system, iMHere (interactive mobile health and rehabilitation; Figure 1), is a platform consisting of a smartphone app with a suite of modules aimed at managing various medical conditions, a Web-based clinician portal, and a communication system connecting patients with clinicians and caregivers. Some specific modules within the iMHere self-management app target medication management (MyMeds), skin integrity (SkinCare), bowel management (BMQ), bladder self-catheterization (TeleCath), and mental health (Mood).

The first version of iMHere (v1.0) did not offer sufficient accessibility—and, thus, usability—to persons with intellectual disabilities or dexterity impairments. Our prior work [15] revealed that the personalized user interface (UI) design may improve accessibility. In addition, this work generated a list of design requirements for the next iteration of the software. These design requirements were as follows:

1. Using simple and common words to ensure the readability and understandability of the text to help users better understand the app by simplifying the cognitive processes needed for completing tasks.
2. Using shortcuts in navigation to make a given task easier to complete.
3. Reducing the number of touches to reduce the burden of navigation and text entry.
4. Implementing contrasting colors between the text and background, as well as adding text-shadows, to enhance the contrast and improve readability.
5. Providing a short, one-sentence reminder offering directional guidance to prevent mistakes related to task procedures.
6. Using large icons and buttons to improve accessibility, especially for users with dexterity impairments.
7. Implementing colors to indicate the status of medications to let users know whether or not a medication is scheduled.
8. Separating the modules by color to easily signal which module is in use.
9. Using color-coded body parts on a map of the body to help users correctly specify the location of a skin problem.
10. Hiding the unused modules from the iMHere dashboard, selecting text display size, and changing contrast and display theme to make the system more personalized.

In general, users expressed a desire to have a simpler app that is easy to understand and physically use [15,16]. Because an mHealth app is a user’s data point of input, such accessibility is essential for users in performing their self-management-related activities and reporting or communicating with their clinicians.

Identifying patient needs and preferences with respect to using an iMHere app delineates only one step in the process of creating greater levels of accessibility. We believe that the accessibility of mHealth can be enhanced with user-centered design and implementation. Better accessibility of smartphone apps may benefit some of the 4.04 million adults in the United States with dexterity impairments [17] whose medical problems can be addressed with iMHere.

This study aims to design accessible features in the iMHere self-management app for persons with intellectual disabilities and dexterity impairments. We hypothesized that use of the redesigned app would result in significantly improved usability measures compared with the use of the original app. Results from this study will be used to develop a new version of the software.

## Methods

### Development Method

**Figure 2:**
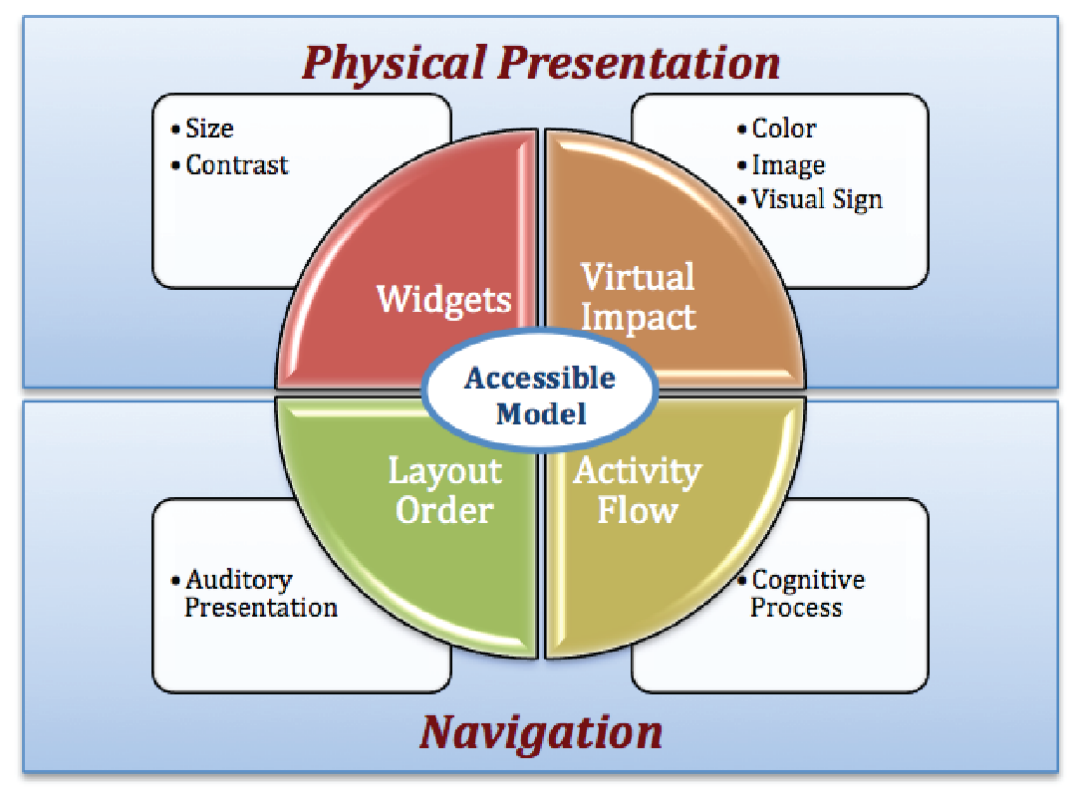
Four elements of the user interface.

An earlier evaluation study [15] suggested that possible accessibility issues could be mitigated with better app design and development. We believe that the approach to designing an accessible interface involves working with two primary UI components: physical presentation and navigation (Figure 2). The physical presentation includes the following:

- *Presentation of widgets*: Focuses on the size and contrast of text and the use of buttons. The size of the widgets (icons) and text and the contrast can be adjusted to users’ preferences.
- *Visual impact*: Focuses on the use of charts, images, and visual cues.

Navigation refers to activity flow and layout order in terms of effectiveness. Simple navigation is important for all users, but especially important for people with dexterity or cognitive impairments. The proposed design approaches the app’s accessibility in terms of navigation from the following aspects:

- *Activity flow*: Focuses on the cognitive process, on providing straight-line experiences for a user to complete a task. Good activity flow means the user is able to effectively and efficiently locate the needed information in the smartphone app.
- *Layout order*: Focuses on the presentation of individual screens. Placing related information in close proximity makes it easier for a user to understand the presented information. In addition, having consistent layouts across the modules within the app provides a smooth learning curve for users.

### Usability Study Method

After the development of new accessibility features, a usability study was conducted. Inclusion criteria were as follows: users must have participated in the prior usability study [15], be aged 18-55 years, have dexterity issues in the fingers or hands, have an active condition or past history of skin breakdown from using a wheelchair or having insensate areas of skin, and be taking at least one prescription or nonprescription medication. Exclusion criteria were as follows: users having any problem in vision, hearing, or conversation that completely precluded the use of a mobile phone.

Manufacturers have moved to replace the physical keyboard with virtual or soft keys to reduce the size and weight of smartphone devices. To not leave PwDs behind in the area of smartphone touch screen technologies, this research examined the use of apps on a smartphone with virtual or soft keys (touch screen). Specifically, this research utilized Samsung Galaxy, a lightweight, touch screen-enabled, slate format Android smartphone with no physical keyboard (dimensions: 4.82 in×2.53 in×0.55 in; weight=5.5 oz); this screen size is smaller than the current standard screen size, which is ≥5.5 in.

The Institutional Review Board of the University of Pittsburgh approved this study. All participants were asked to provide informed consent. We enrolled all nine participants (9/9, 100%) from the prior evaluation study [15] were enrolled. All of these individuals could be classified as experienced participants but had abstained from using the iMHere app for >4 months before participating in this study. This abstinence is aimed at minimizing the potential learning effects that could ostensibly carry over from the previous experiences.

The Purdue Pegboard Assessment, a popularly utilized diagnostic tool for measuring the movements of a person’s fingers, hands, and arms, was used to measure the baseline for participant dexterity levels [18-22]. We used 4 tests from the Purdue Pegboard Assessment in this study. The assessment comprised 4 tests with 30-second intervals using the right hand, left hand, and both hands, yielding a composite score of “right+left+both hands.” During these tests, participants were asked to pick up pins, collars, or washers from the top of the board and drop them into the peg holes. The score for each test was based on the total number of pins, collars, or washers dropped into the holes correctly. The “right+left+both” hand score was used as the basis for evaluating a participant’s dexterity, with lower “right+left+both” hand scores indicating a higher degree of dexterity impairment.

This study focused on two specific modules within the iMHere self-management app: MyMeds for medication management and Skincare for skin monitoring and reporting of skin breakdown. These two modules were selected not only on the basis of their critical importance to self-management for individuals with chronic conditions like SB and SCI but also for their relative complexity.

A 1-week field trial was completed, in which participants were asked to use the two modules in their daily lives. Afterwards, a laboratory-setting evaluation and in-depth interview were conducted. A “think-aloud” protocol [23] requires participants to verbalize their thoughts as they attempt to complete the tasks, thereby allowing investigators to identify further usability or accessibility issues that need to be addressed. The “think-aloud” method required participants to describe, in words, what they see, think, do, and feel while performing the tasks needed to navigate through the two modules. The following tasks were included in the laboratory test:

- *Task 1*: Scheduling a new medication alert; this includes searching for and finding the correct medication as well as setting up a medication schedule.
- *Task 2*: Modifying a medication reminder, which includes changing the alert time for a medication.
- *Task 3*: Responding to a medication alert, which includes indicating whether the participant took a particular medication.
- *Task 4*: Scheduling an alert to remind oneself to check the skin for any issues or problems.
- *Task 5*: Responding to a skincare reminder, which involves taking a picture and describing any dermatological issues through a series of survey questions.
- *Task 6*: Setting personalized configurations for UI presentations, including choosing a preferred list of modules, modifying the reading size of text, and choosing the size of onscreen buttons.

The researcher first explained the tasks to the participant until he or she understood the details of each activity (approximately 15 min). Once the participant was well informed of his or her expectations in performing the tasks, a quantitative evaluation was performed, and the following usability measures were collected:

- *Importance ranking*: Participants were asked to rate the new accessibility features on a scale from 1 to 10 (1=most important feature; 10=the least important feature).
- *User effort*: The minimum number of times the participant needed to touch the screen to complete all tasks.
- *Individual task time*: Average time to complete a specific task.
- *Overall task time*: Average time to complete all tasks.
- *Error rate*: The number of errors or mistakes committed during all tasks.
- *Usability*: Participants were asked to complete a modified version of the Telehealth Usability Questionnaire (TUQ) [24,25]. The TUQ is a qualitative survey covering the following factors—usefulness, ease of use and learnability, interface quality, interaction quality, reliability, and satisfaction and future use [24,25]. In assessing these factors, the TUQ utilizes a 7-point Likert scale (with a value of 1 as least usable and 7 as most usable). An overall average score and individual factor scores were calculated.

An in-depth interview was subsequently conducted to gather participant feedback and impressions regarding the iMHere app.

### Statistical Analysis

All the data collected from this study were uploaded to SPSS (IBM Corp. Released 2016, IBM Statistics for Windows, Version 24.0, Armonk, NY: IBM Corp) for statistical analysis. The sum and average task completion times were utilized to measure participants’ performance levels. Error rate was calculated as the number of errors or mistakes divided by the total of steps taken to complete tasks. SDs were calculated to reveal any possible dispersion patterns. The results from the previous evaluation study of the originally designed iMHere app [15] were used here for comparison.

Because our sample size was smaller than 50, Shapiro-Wilk test was used to determine whether the data were normally distributed. As all data were normally distributed, paired *t* tests were utilized to evaluate differences between the original and new app with regard to usability measures. Statistical significance was set at *P*<.05.

## Results

### Backgrounds of Participants

Of the 9 participants from the earlier evaluation study [15], 3 were lost due to follow-up issues (ie, changed phone number or had relocated). Overall, 6 participants completed this study. Of all participants, 5 had SB and 1 had SCI. All participants with SB had some degree of cognitive impairment related to shunted hydrocephalus.

All 6 participants were right-hand dominant and all met the inclusion and exclusion criteria. All individuals with SB had spinal lesion levels at the low thoracic or lumbosacral levels. The participant with SCI had a cervical lesion level.

As shown in Table 1, all participants’ “right+left+both” hand scores were below −2 SD from the mean score of general factory workers (46.76−2 SD=38.68) [26]. Participants 1, 5, 6, and 7 tried picking up pins using both hands and dropping the pins in the holes at the same time to speed up their performance. This led to scores for the “both-hand test” that were around the mean of general factory workers at 16.01. Participant 8 had experienced a traumatic SCI (C5) resulting in minimal movement of the arms, a slight movement of the thumb and index figure, and an inability to hold or pick up objects. In addition, participant 8 was unable to perform the pegboard assessment test, but could access a smartphone either using the side of the fifth digit or a stylus mounted to a custom orthosis.

### Development Results

Table 2 shows the number of individuals assigning high (1-3, very important), medium (4-7, important but not essential), and low (8-10, less important) ranks for each newly developed accessibility feature.

**Table 1:** Background of participants (P).

Question	P01	P03	P04	P05	P07	P08
Age (in years)	36	27	25	20	33	22
Highest education	Graduate	High school	High school	High school	Undergraduate	Graduate
Gender	Female	Male	Male	Male	Female	Male
Regular phone versus smartphone	Regular	Regular	Smartphone	Smartphone	Smartphone	Smartphone
Physical keypad versus touch screen	Physical	Physical	Touch	Touch	Touch	Touch
Mobile phone experience (in years)	0-2	>5	>5	>5	>5	>5
Daily use (in minutes)	>60	>60	>60	>60	>60	>60
Pegboard score right+left+both	33.00	27.00	23.67	36.33	37.00	0.00

**Table 2:** Importance ranking.

#	Features	Number of individuals assigning ranks		
		Ranks 1-3	Ranks 4-7	Ranks 8-10
1	Customized app list	2	2	2
2	Customized text display size	2	1	3
3	Customized theme	0	2	4
4	Customized button size	2	3	1
5	Customized keyboard	2	2	2
6	Ability to take a picture of a pill or med bottle	2	4	0
7	Color-coding	1	2	3
8	Text guide	2	4	0
9	Voice guide	4	1	1
10	Short cut for navigation	2	2	2

**Figure 3:**
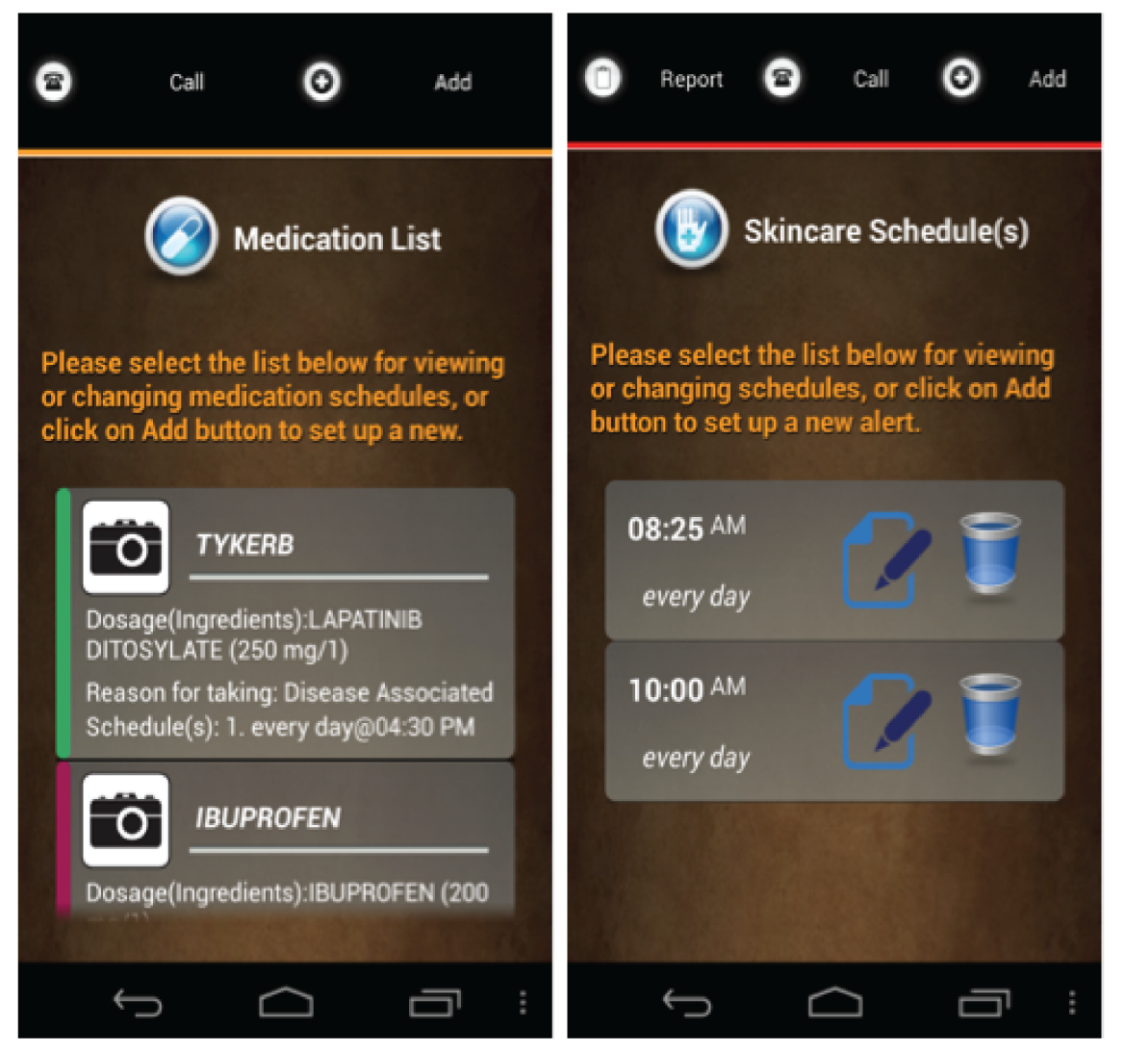
Screenshots of the use of color-coding at the app level. (Source: Created by the authors).

**Figure 4:**
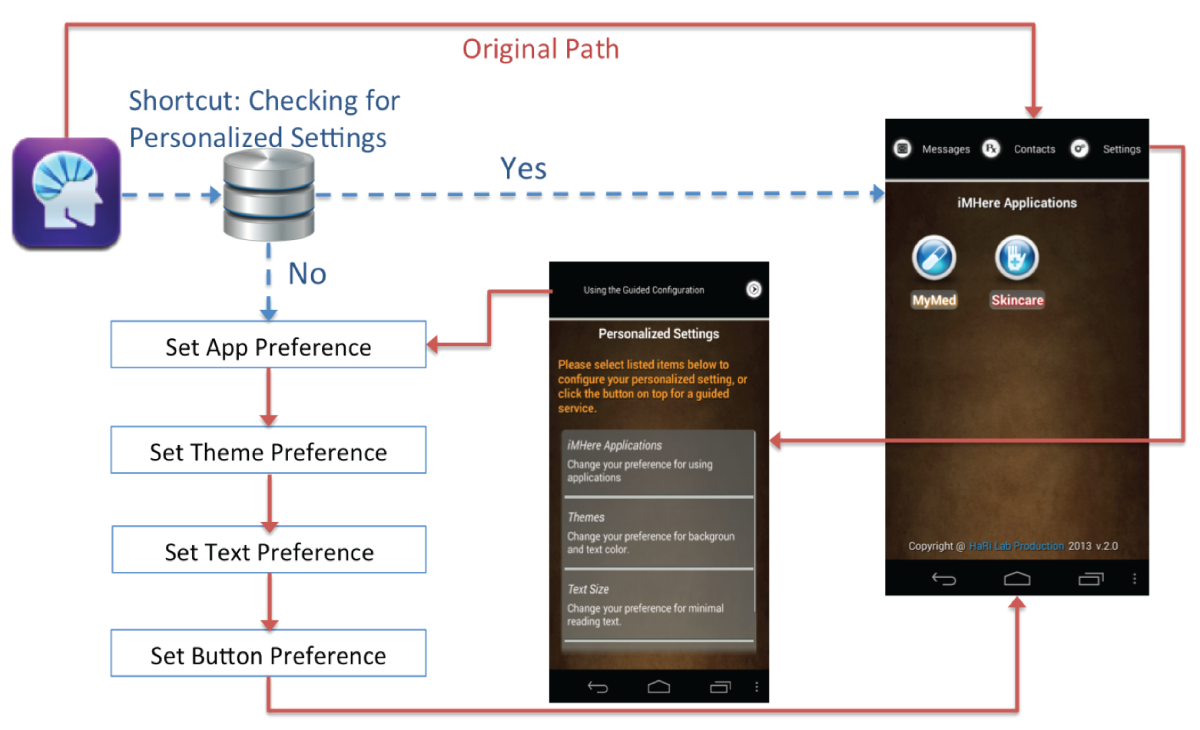
Navigation for personalized configuration.

1. *Customized app list*: This feature provides the ability for a user to hide or show a selected module from the home screen. Overall, 67% (4/6) participants thought that the first feature was important to hide the TeleCath and BMQs apps because they did not need to catheterize the bladder (TeleCath) or perform bowel management (BMQs).
2. *Size of display text*: A user can specify his or her minimal and comfortable reading size. This display size is then used as the foundation for all other configuration parameters for text display in iMHere modules. Overall, 50% (3/6) participants thought that using customized text size was important; participants 1, 3, and 8 ranked this feature 2, 3, and 4, respectively.
3. *Customized theme*: The feature allows the user to select his or her preferred background and text color. Although all participants reported liking this feature, 67% (4/6) participants, that is, participants 1, 4, 7, and 8, thought it to be unnecessary for improving the accessibility of the modules. These participants ranked this feature as 10, 10, 9, and 8, respectively.
4. *Customiz* *ed* *button size*: The system asks the user to press his or her index finger on the screen to record his or her fingertip size. This touch size was used as the minimum target size for buttons or icons in the accessible design. Overall, 83% (5/6) participants thought this feature was important. Participants 4 and 8—notably individuals who presented with a higher degree of dexterity impairments—ranked it as the second most important accessibility feature.
5. *Customized keyboard*: A customized keypad with softer keys, larger key sizes, and preconfigured characters was designed to reduce the number of required touches on the smartphone screen. When using the customized keypad to enter “2 tablets,” of a medication, for instance, the users would touch “2” and “tablet.” This 2-touch entry can be contrasted with the 8-touch entry necessitated by using a traditional keypad for text entry. Overall, 67% (4/6) participants identified this feature as important for them. In particular, participant 8 (with severe dexterity impairments) ranked the customizable keyboard as the most important feature.
6. *Ability to take a picture of a pill or bottle*: This feature provides the ability for a user to take a photo of a pill or medication bottle and upload it into his or her medication schedule. With this feature, a user can “double verify” the medication is correct by comparing it with a picture before taking his or her prescribed dose. Overall, 33% (2/6) participants ranked this feature as one of the most important.
7. *Color-coding*: As suggested by participants in the earlier evaluation study [15], color-coding was utilized in the new design to help a user navigate within the modules. For example, the title for the SkinCare module has been highlighted in red, and all screens under the SkinCare module now have a red bar to remind the user which module is being used (Figure 3). Participant 5 indicated that this feature was very important for him, as it provided a way to remember which module he was using. This participant ranked the color-coding feature as 3. Participants 3 and 6 thought this feature was important but might not be essential. Participant 7 thought this feature might be beneficial to users with intellectual disabilities.
8. *Text guidance*: Text containing self-training instructional notes is displayed on the screen and highlighted in a particular color (such as orange in Figure 2). Participants 3 and 4 ranked the text guidance as a very important feature to them, ranking this feature as 2 and 3, respectively. The remainder thought the text guidance was important but not essential, providing respective rankings of 4 and 6.
9. *Voice guidance*: Using text-to-speech technology, users can listen to text guidance as audio output. Participants 4, 5, 7, and 8 (ie, 4/6, 67%, participants) thought this voice guidance ability was important, ranking it as 3, 1, 1, and 3, respectively.
10. *Navigational short cut*: The newly designed app allows for personalization on the level of navigation. For example, the system checks the database for personalized settings first (Figure 4). If no personalized settings are found, the system will then lead the new user to set his or her preferences before going to the home screen (a list of modules). Overall, 33% (2/6) participants indicated that the ability to create shortcuts in navigation was very important to them. Participants 1 and 5 ranked this feature as 1 and 3, respectively, while participants 5 and 8 thought this feature was important but not essential, ranking it as 4 and 7, respectively.

### Usability Study Results

Table 3 displays user effort results. Overall, user effort to complete all tasks was reduced by an average of about 25% in the redesigned modules. A lower average number of touches was needed for completing tasks with the redesigned modules (mean 7.20, SD 4.82) than with the original modules (mean 10.80, SD 8.04), but this difference was not statistically significant (*t*_4_=2.25; *P*=.09).

Table 4 shows individual task time results. The average time to complete individual tasks was reduced by just over 50% in the redesigned modules. Participants spent the most time on tasks that required scheduling a medication or reporting a new skin problem. Particularly, task 3, responding to a medication alert, showed only a small improvement in completion time (7.7%).

**Table 3:** User effort: minimum number of screen touches to complete a task.

Tasks	Original modules (n)	Redesigned modules (n)	Difference (n)	Change in effort (%)
Schedule med alert	20	11	−9	−45
Modify med alert	9	6	−3	−33
Respond to med alert	1	1	0	0
Schedule a skin check	6	5	−1	−17
Report a new skin problem	18	13	−5	−28
Average effort	10.80	7.20	−3.60	−24.60

**Table 4:** Individual task time: average time needed to complete individual tasks.

Task #	Tasks	Original modules (seconds), Mean (SD)	Redesigned modules (seconds), Mean (SD)	Time difference	
				Seconds	Percentage
1	Schedule medication alert	203.2 (122.8)	89.2 (49.5)	−114.1	−56.1
2	Modify medication	61.8 (43.6)	18.8 (5.6)	−43.0	−69.5
3	Respond to medication alert	2.9 (1.4)	2.7 (1.0)	−0.2	−7.7
4	Schedule skin check	42.8 (31.3)	15.7 (4.5)	−27.1	−63.4
5	Report new skin problem	147.9 (87.1)	61.3 (22.5)	−86.6	−58.5
	Average task time (seconds)	91.72 (57.24)	37.54 (16.62)	−54.20	−51.04

**Table 5:** Telehealth Usability Questionnaire (TUQ) scores and overall task time for each participant.

Parameter		P01	P03	P04	P05	P07	P08
**TUQ score, mean (SD)**							
	Original modules	6.55 (0.7)	6.35 (0.9)	5.55 (0.9)	6.10 (0.7)	6.35 (0.5)	5.60 (1.1)
	Redesigned modules	6.90 (0.3)	7.00 (0.0)	6.89 (0.3)	7.00 (0.0)	6.60 (0.5)	6.70 (0.5)
**Overall task time in seconds, mean (SD)**							
	Original modules	127.20 (108.7)	68.40 (59.4)	79.40 (73.4)	104.00 (114.5)	44.60 (40.9)	66.60 (58.6)
	Redesigned modules	33.50 (24.6)	38.00 (22.8)	38.83 (29.9)	53.00 (69.7)	22.17 (21.7)	26.50 (27.3)

This small increase may be attributed to the fact that this task involved only a single click on the alert screen for both the original and redesigned modules. The average time to complete individual tasks was higher using the original modules (mean 91.72, SD 81.79, seconds) than using the redesigned modules (mean 37.54, SD 36.32, seconds), but this difference was not statistically significant (*t*_4_=2.64; *P*=.06).

Table 5 shows the average time in seconds for each participant to complete all 5 tasks and TUQ scores. A significantly lower average time for users to complete all 5 tasks was observed with the use of the redesigned modules (mean 35.33, SD 10.83, seconds) than with the use of the original modules (mean 81.70, SD 29.51, seconds; *t*_5_=−4.52; *P*=.006). Significantly higher overall average TUQ scores were observed with the use of the redesigned modules (mean 6.85, SD 0.16) than with the use of the original modules (mean 6.08, SD 0.42; *t*_5_=4.39; *P*=.007).

As shown in Table 6, the error rate using the redesigned modules (mean 0, SD 0) was significantly lower than that using the original modules (mean 8.51, SD 5.55; *t*_5_=3.76; *P*=.01). In fact, no participants made errors using the redesigned modules.

**Figure 5:**
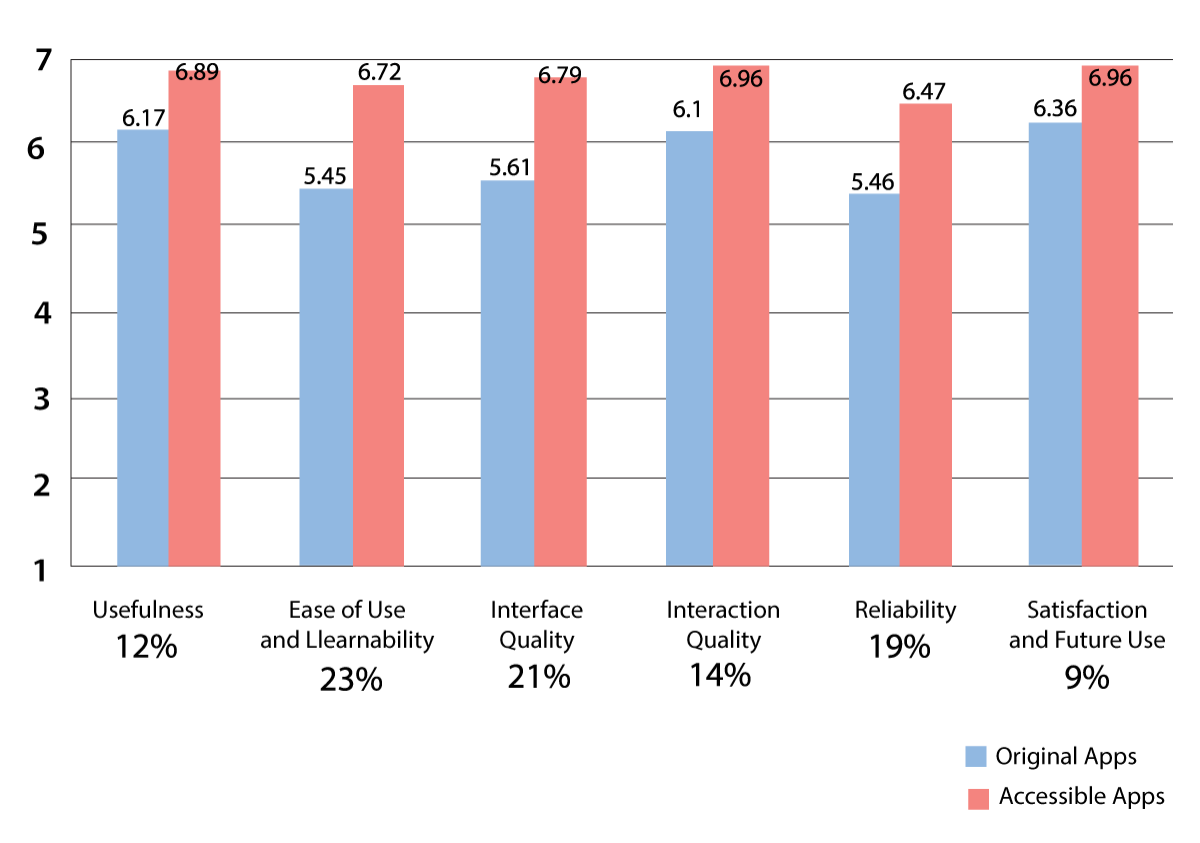
Telehealth Usability Questionnaire factors, scores, and percent increase.

When comparing the average subscale scores for the 6 individual domains of the TUQ with the subscale scores in the earlier evaluation study [15], usability improved significantly from the original app (mean 5.86, SD 0.40) to the redesigned app (mean 6.80, SD 0.19; *t*_5_=−8.81; *P*<.001). As shown in Figure 5, pronounced improvements were noted for the factors “ease of use and learnability,” “interface quality,” and “reliability” (>15% improvements).

**Table 6:** Comparison of the error rates.

Participant	Original modules (%)	Redesigned modules (%)
P01	7.17	0.00
P03	0.00	0.00
P04	16.08	0.00
P05	5.75	0.00
P07	10.00	0.00
P08	12.08	0.00
Average	8.51	0.00

## Discussion

### Principal Findings

A smartphone is an ideal tool for implementing self-management programs for PwDs [27], but it does pose accessibility challenges. The size of the screen and the mobile device itself is the main obstacle to accessibility [28-30]. The small screen becomes easily cluttered when a designer wishes to fill the space with attractive text, images, and widgets [30]. This small size of the screen leads to an issue with usability [31] because it is difficult for users to read [32]. The small target or touch size, low contrast, and inappropriate text size presented on a small screen might be problematic for users with visual or dexterity problems to access [33-35]. In addition, unnecessary options and functions create difficulties for users with intellectual disabilities to understand the process, as well as to recall procedures [32].

**Figure 6:**
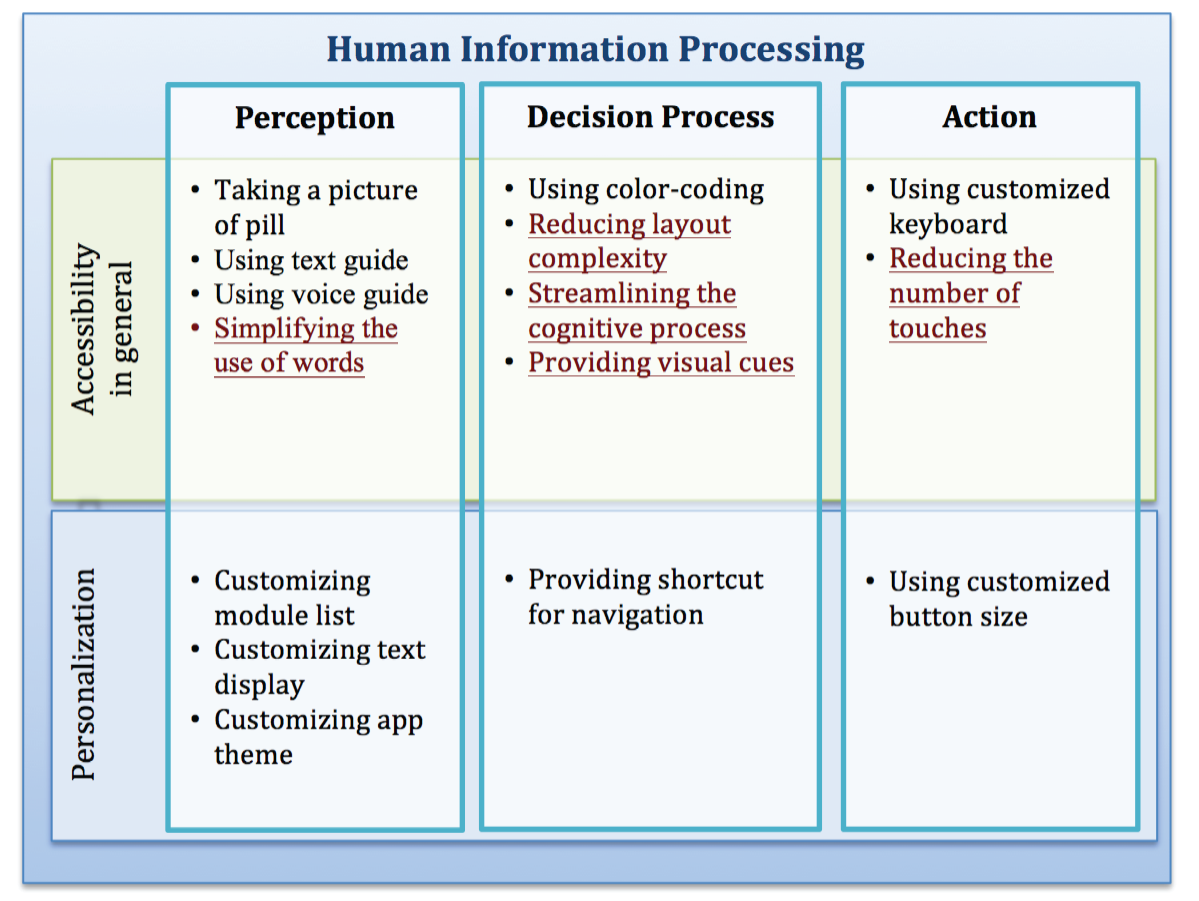
Accessibility strategies.

Some of the abovementioned accessibility issues can be mitigated with design and development of a better UI. The results of this study and our prior studies [15,16] reveal strategies important to improving accessibility of smartphone apps. These strategies are presented in Figure 6, organized according to the different stages of human information processing. The text underlined in Figure 6 indicates the accessibility strategies that are important for general users; the other text indicates important features for persons with dexterity impairments.

Some features such as the customized app list, reading size, theme, and button size made the system simpler and more conducive to personal use. Particularly, the small target size of icons or buttons presented a problem for users with dexterity impairments due to the decreased strength and sensation in their fingers.

The redesign implemented in this study was based on the findings that the size of buttons has a significant impact on usability. Chen et al found that users without disabilities plateaued with a minimal button size of 20 mm and users with disabilities plateaued at 30 mm [36]. Colle and Hiszem found that 20 mm^2^ buttons resulted in optimal user performance for younger participants [37], while Jin et al suggested a button size of 19.05 mm for elderly users [38]. Monterey Technologies Inc recommends the button size to be at least 19.05 mm [39]. In addition, Apple recommends a minimum target size of 44 pixels wide and 44 pixels long (by 11.64 mm) [40]. Notably, all these prior studies assumed a fixed button size.

We introduced the ability to measure the finger or touch size of a user via the smartphone as well as the ability to leverage that measurement toward creating an optimum target button or icon size. This feature is especially beneficial for users with a higher degree of dexterity impairment.

In addition to the abovementioned features, participants also found the following strategies implemented in the redesigned apps to be helpful:

- *Multiple-choice questions in place of text entry*: All participants found that making a selection was easier than entering long lines of text. Text entry, however, should always be an option in the list; if a user selects “other” he or she can then operate the text function and answer the given prompt in more detail.
- *The volume button has been appropriated as the camera button*: Except for participant 8, who was unable to hold a smartphone, all other participants liked being able to use the volume control button to take a picture, especially when taking photographs of a skin wound located in a difficult to reach area.
- *A self-directed questionnaire has been utilized to simplify the cognitive procedures of tasks*: Compared with the regular format, the redesigned modules show only one question at a time. The system automatically proceeds to the next question after a user makes a selection. In this study, 4 of 6 participants indicated that the process flow in the self-directed questionnaire was easier to understand and follow as a result of offering more guidance and fewer functions per screen.

Most notably, the average time to complete tasks in this study was reduced by about 60% in the redesigned modules. Usability of the redesigned apps as measured by TUQ showed a significant increase. Pronounced improvements were particularly noted for the factors “ease of use and learnability,” “interface quality,” and “reliability.” Finally, the redesigned modules were able to eliminate all errors that occurred during use of the original modules.

A surprising finding of this study was the degree of dexterity impairment identified in participants with SB. All participants had spinal lesion levels in the low thoracic or lumbosacral areas, which means that there was no paralysis of the arms or hands. Impairments in fine motor control in SB are thought to be due to the abnormal organization of the cerebral cortex [41,42]. All participants with SB, however, had pronounced impairments in dexterity, measured as >2 SDs below normative values. Little is known about the extent of fine motor control problems in SB and how it affects the use of mHealth technologies.

### Limitations and Future Studies

Only a limited number of participants were involved in this study of the redesigned iMHere modules. The development and usability study follow the iterative design [43], which consists of a cycle process of prototyping, testing, analyzing, and refining a system. This study is at the later stage of the iterative cycle that follows previous studies [15,16]. By limiting the evaluation to participants from the earlier studies, we were able to probe deeper into the usability of the fundamental structure of the mHealth apps and to find majority of the usability problems [43].

The results of this study should be viewed with the nature of participants’ impairments in mind. The next study should include more participants with varying levels of dexterity impairments— as well as a wider range in the diagnoses underlying these impairments—to better assess the overall acceptance and preference of the redesigned modules. In addition, more studies into the various degrees of dexterity impairments in individuals with SB, and the effect(s) of these impairments on the use of mHealth technologies, are warranted. Furthermore, future studies are warranted on the usability of the iMHere clinician portal and caregiver app—work that is conducted in parallel with studies performed on the patient app.

### Conclusion

The accessibility standards and guidelines such as the Web Content Accessibility Guidelines 1.0 [44] and 2.0 [45] are mainly aimed at improving the general accessibility of the Web, not specifically of smartphone apps. The cross-platform technology for developing smartphone apps, which is based on Web technology, is increasingly popular. We plan to implement the strategies and accessibility principles in this study to the cross-platform app development environments that is based on Web technology in our future studies.

This study proposes a design and developmental model to approach accessibility through two primary elements of UI: physical presentation and navigation. A usability study showed that the effectiveness and efficiency of, and user satisfaction with, the redesigned modules significantly improved after implementing accessibility strategies into the UI design. As the results suggested, the meaningful presentation and navigation flow also helped us achieve a smoother activity flow during task completion. By extending the concept of personalization to navigation and task flow, the efficiency of users’ performance could be significantly improved.

The aforementioned accessibility strategies and features could be used for other developers to design and develop smartphone apps. This paper focuses on the general principles of accessible mHealth design. Most of the UI elements can be implemented as an accessibility personalization setting of an mHealth app. We plan to implement accessibility personalization feature in our future mHealth developments.

## Abbreviations

iMHere: interactive mobile health and rehabilitation
mHealth: mobile health
PwDs: persons with disabilities
SB: spina bifida
SCI: spinal cord injuries
TUQ: Telehealth Usability Questionnaire
UI: user interface
